# Editorial: Exosomes: Message in a vesicle

**DOI:** 10.3389/fphar.2022.1018928

**Published:** 2022-09-08

**Authors:** Suman Dutta, Satish Balasaheb Nimse, Kendall Van Keuren-Jensen, Gal Bitan

**Affiliations:** ^1^ Department of Neurology, David Geffen School of Medicine, University of California, Los Angeles, Los Angeles, CA, United States; ^2^ Institute of Applied Chemistry and Department of Chemistry, Hallym University, Chuncheon, South Korea; ^3^ Neurogenomics Division, The Translational Genomics Research Institute, Arizona, AZ, United States; ^4^ Brain Research Institute, University of California, Los Angeles, Los Angeles, CA, United States; ^5^ Molecular Biology Institute, University of California, Los Angeles, Los Angeles, CA, United States

**Keywords:** extracellular vesicle (EV), intercellular communication, biomarker, drug delivery vehicle, therapeutics

**FIGURE 1:**
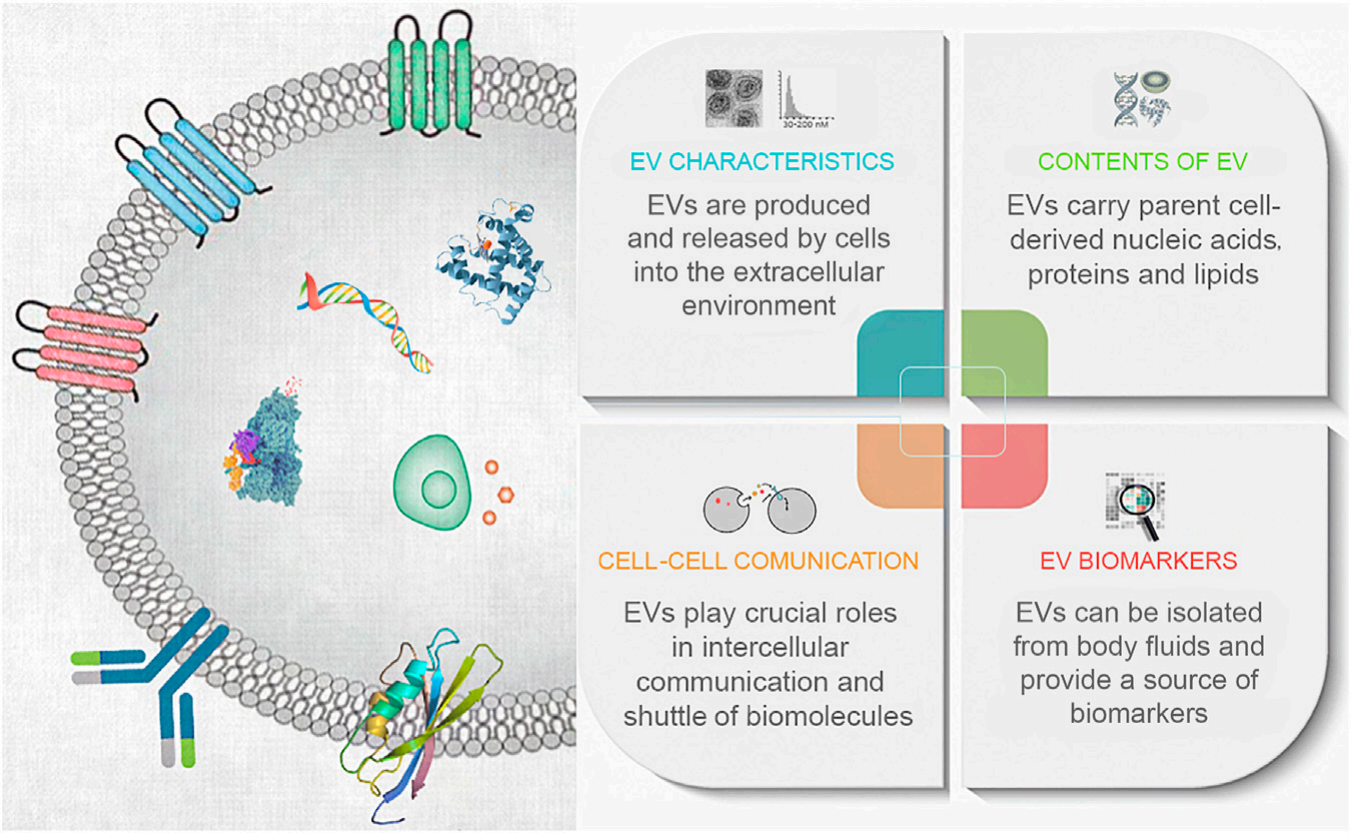
An infographic overview of extracellular vesicles.

Exosomes are a class of extracellular vesicles (EVs) comprising a heterogenous population of biological nano-vesicles enclosed by a lipid bilayer (Yan et al., 2019). All eukaryotic cells produce and release EVs into the surrounding extracellular space, including exosomes. Because the term ‘exosome’ refers to a specific biogenesis pathway that produces some EVs (Lötvall et al., 2014), and once they leave the cell, it is difficult to separate exosomes from other similar types of extracellular vesicles, we refer to all extracellular vesicles as EVs in this editorial. EVs carry various cargoes including nucleic acids, such as miRNA and mRNA, proteins, and lipids. Although EVs originally was thought to be a disposal mechanism of unwanted biomaterials, it became evident in recent years that they play a crucial role in intricate signaling pathways and intercellular communication (Figure 1). Collaborative efforts across the globe have helped elucidate the pathway of EV biogenesis and their involvement in several human diseases. This Research Topic is a collection of original articles, review articles, and one brief research report that highlight current knowledge on different isolation and characterization methods for EVs, their roles in intercellular communications, therapeutic applications, potential as a source of biomarkers, and their use as a vehicle for drug delivery. In total, the Research Topic contains sixteen papers by experts in the corresponding fields describing and discussing enthralling breakthroughs over the past several years in the EV research field.

Shpigelman et al., at the University of California, San Diego, CA, United States, are reporting a pioneering study developing a reporter cell line for quantitative measurement of EV release from antigen-presenting cells (APCs) in a phenotypic high-throughput screening format. They show that the new, dual-reporter cell line exhibits robust responses and has the potential to become an excellent research tool for the screening of compounds that induce or inhibit EV release by APCs. An article by Shukla et al., at the University of California, San Diego, CA, United States, aimed at identifying small molecules that could induce EV release in a human monocytic leukemia THP-1 reporter cell line. They described a process of screening the EV release that could add a new dimension to structure-activity relationship studies of vaccine adjuvant activity. A study by Olivero et al., at the University of Genoa, Italy, addresses the important question of whether exosomes are actively released from presynaptic nerve terminals. Their findings provide strong evidence supporting the release of EVs from presynaptic structures and indicate an increased EV release upon exposure of synaptosomes to a depolarizing stimulus.

Alsop et al., from the group of one of our Research Topic Editors, Dr. Kendall Van Keuren-Jensen at the Translational Genomics Research Institute, Phoenix, AZ, United States, together with multiple collaborators, have generated an atlas of small RNAs from 30 different tissues and three different blood cell types. They analyzed various tissues for the enrichment of small RNA sequences, assessed their expression in different human biofluids (saliva, urine, CSF, and plasma) in cell-free circulation as well as from EVs (Kalani et al., 2020), and developed an online tool providing information about extracellular-RNA sequences found in different biofluids and tissues. The authors made the tool, which shows tissue-specific elevation of extracellular RNA sequences and their abundance in biofluids, freely available online. Sandau et al., at Oregon Health and Science University, Portland, OR, United States, with several collaborators, sought to understand the effect of Alzheimer’s Disease (AD) on the miRNA cargo in cerebrospinal fluid-derived EVs and to determine if AD risk factors have an impact on EV-associated miRNA expression patterns. Their study showed that APOE-ε4 and female sex, two known risk factors for the disease, influence EV-associated different miRNA levels in the CSF of patients with AD. Bilousova et al., at UCLA, CA, United States, led a multi-omics study analyzing putatively microglia-originating small EVs from cryopreserved human brain tissue in search of novel biomarkers for Alzheimer’s Disease. The analysis included lipids, proteins, and miRNAs in microglia-originating small EVs from human brain tissue and indicated the release of neuronal and myelin materials via EVs. Another important finding was a significant decrease in docosahexaenoic acid levels in AD, which potentially could be used as a biomarker. A review article by Ghosh and Ghosh from the Indian Institute of Technology Jodhpur, India, described in detail the role of small EVs as pathogenic agents transmitting toxic protein forms that propagate neurodegenerative disorders throughout the CNS. It is becoming increasingly evident how small EVs can be harnessed in identifying neurodegenerative disease biomarkers (Hornung et al., 2020; Dutta et al., 2021).

A review article by Hu et al., from Guangdong Medical University, Dongguan, China, discussed the potential of small EV-associated cargo as a source of novel biomarkers for cancer. The authors gathered useful information regarding the clinical applicability of EV-associated proteins based on which they suggest that the advancement of EV isolation techniques and characterization strategy will play a significant role in diagnosis as well as immunotherapy of cancer. Another detailed review by Holcar et al., from the University of Ljubljana, Slovenia has summarized major contaminants of blood-derived EVs and thoroughly discussed how different EV isolation methods might affect the EV yield and data interpretation of downstream experiments. The authors reiterated the importance of developing methods for isolation of pure subpopulations of EVs to which the origin of the parent cell type can be attributed and argued that such development will help understand the functions of EVs in a definitive way.

A timely study by Pesce et al., from the National Institute of Molecular Genetics (INGM), Milan, Italy, demonstrated that EVs isolated from the plasma of patients with COVID-19 harbor SARS-CoV-2-related protein materials. They observed that EVs recovered from patients with mild symptoms effectively modulated antigen-specific CD4-positive T-cell responses. The study also suggested that the patient-derived EV-associated proteins not only induced an immune response, but also the EV cargo could help identify the pathological state of patients in the acute phase of the disease.

Moros et al., at the Universidad de Zaragoza, Zaragoza, Spain, and Istituto di Scienze Applicate e Sistemi Intelligenti, Pozzuoli, Italy, isolated EVs from the freshwater cnidarian polyp *Hydra Vulgaris* and investigated their biochemical, morphological, and functional characteristics. The analysis showed that EVs released by this species transfer protein and major components of axial patterning, modulating foot and head regeneration. A review article by Fang et al., China Agricultural University, Beijing, China, and Dr. Brett M. Tyler Oregon State University, Corvallis, OR, United States compiles information regarding recent research on the biogenesis of EVs and their pathobiological functions in organismal communication of microbes. The authors highlighted key questions related to the transfer of genetic materials between species which requires further exploration in this direction.

The delivery of therapeutics across the blood-brain barrier for brain disorders has remained challenging despite recent progress (Terstappen et al., 2021). A study Kutchy et al., at the University of Nebraska Medical Center, Omaha, NE, United States, and collaborators in Grenada and China showed that EVs could be used as a vehicle for the effective delivery of drugs to the brain. The authors investigated the biodistribution of ultra-small, iron oxide-labeled, mouse astrocyte-originating EVs after intranasal perfusion in mice and suggested that engineered EVs could be utilized for targeted delivery of therapeutics into several organs including the brain. In another review article, Zheng et al., from Fudan University, Shanghai, China, summarized the current research advances on the involvement of EVs in the pathogenesis, diagnosis, and treatment of the autoimmune disease, systemic lupus erythematosus (SLE) and lupus nephritis (LN). In this article, the authors have discussed the role of several immune cell-originating EVs in the regulation of the innate immune responses and highlighted the necessity of further research to fully understand the involvement of EVs in the pathophysiological mechanisms of SLE and LN.

While EVs have been extensively studied in the field of cancer (Xu et al., 2018) and some selective neurodegenerative disorders (Hill, 2019; Hornung et al., 2020), their involvement in bone regeneration is limited. Kang et al., at the University of Illinois, Chicago, IL, United States, studied the effects of the inflammatory cytokines on mesenchymal stem cell-derived EV-mediated immunomodulation. They concluded that TNFα preconditioning of human mesenchymal stem cells results in the generation and release of EVs that can alter the phenotype of macrophages, both *in vitro* and *in vivo*. Corsello et al., at the University of Texas Medical Branch at Galveston, TX, United States, investigated the presence of innate immune mediators, including interferons in EVs, released from airway epithelial cells infected with the respiratory syncytial virus. The study found significant levels of soluble and EV-associated interferon λ in nasopharyngeal secretions (NPS) and NPS-derived EVs from children with respiratory syncytial virus infections.

Overall, this Research Topic covers broad aspects of extracellular vesicle biogenesis, their isolation and characterization strategies, involvement in intercellular communication, and the roles EVs play in different human diseases. The topic also includes articles that investigated and discussed the possibilities of using small EVs as potential disease biomarkers and opportunities for therapeutic applications. These basic, translational, and clinical studies demonstrate the importance and high potential of EVs in human health and diseases including neurodegenerative disorders and cancer, as well as in understanding intercellular communication in low organisms, including microbes and polyps. Future scientific efforts to fully understand the mechanisms of EV-mediated cell-cell communication in health and different disease states are expected to provide critical insights into basic biological processes, development of biomarkers, identification of therapeutic targets, and harnessing EVs as a vehicle for drug delivery.
